# Oral Delivery of Bioactive Glass‐Loaded Core–Shell Hydrogel Microspheres for Effective Treatment of Inflammatory Bowel Disease

**DOI:** 10.1002/advs.202207418

**Published:** 2023-04-24

**Authors:** Yanlun Zhu, Yiwei Wang, Guanggai Xia, Xuerao Zhang, Shuai Deng, Xiaoyu Zhao, Yanteng Xu, Guozhu Chang, Yu Tao, Mingqiang Li, Haiyan Li, Xinyu Huang, Hon Fai Chan

**Affiliations:** ^1^ Key Laboratory for Regenerative Medicine of the Ministry of Education of China School of Biomedical Sciences Faculty of Medicine The Chinese University of Hong Kong Shatin Hong Kong SAR 999077 China; ^2^ Institute for Tissue Engineering and Regenerative Medicine The Chinese University of Hong Kong Shatin Hong Kong SAR 999077 China; ^3^ Shanghai Sixth People's Hospital Affiliated to Shanghai Jiao Tong University School of Medicine 600 Yishan Rd Shanghai 200233 China; ^4^ Cell Therapy and Cell Drugs of Luzhou Key Laboratory School of Pharmacy Southwest Medical University Luzhou Sichuan 646000 China; ^5^ Laboratory of Biomaterials and Translational Medicine Center for Nanomedicine The Third Affiliated Hospital Sun Yat‐sen University Guangzhou 510630 China; ^6^ Guangdong Provincial Key Laboratory of Liver Disease Guangzhou 510630 China; ^7^ Chemical and Environmental Engineering School of Engineering RMIT University 124 La Trobe St Melbourne VIC 3000 Australia; ^8^ Hong Kong Branch of CAS Center for Excellence in Animal Evolution and Genetics 999077 Hong Kong SAR China; ^9^ Center for Neuromusculoskeletal Restorative Medicine Hong Kong Science Park Hong Kong SAR 999077 China

**Keywords:** bioactive glass, inflammatory bowel disease, macrophage, microsphere, oral delivery

## Abstract

Resolving inflammation and promoting intestinal tissue regeneration are critical for inflammatory bowel disease (IBD) treatment. Bioactive glass (BG) is a clinically approved bone graft material and has been shown to modulate inflammatory response, but it is unknown whether BG can be applied to treat IBD. Here, it is reported that BG attenuates pro‐inflammatory response of lipopolysaccharide (LPS)‐stimulated macrophages and hence reduces inflammatory damage to intestinal organoids in vitro. In addition, zein/sodium alginate‐based core–shell microspheres (Zein/SA/BG) are developed for oral delivery of BG, which helps prevent premature dissolution of BG in the stomach. The results show that Zein/SA/BG protects BG from a gastric‐simulated environment while dissolved in an intestinal‐simulated environment. When administered to acute and chronic colitis mice model, Zein/SA/BG significantly reduces intestinal inflammation, promotes epithelial tissue regeneration, and partially restores microbiota homeostasis. These findings are the first to reveal the therapeutic efficacy of BG against IBD, which may provide a new therapeutic approach at low cost for effective IBD treatment.

## Graphical abstract

This study demonstrates that bioactive glass (BG), a clinically‐approved bone graft material, can be applied to treat inflammatory bowel disease when delivered in zein/sodium alginate (SA) core–shell microspheres. When administered to acute and chronic colitis mice model, Zein/SA/BG significantly reduces intestinal inflammation by modulating macrophage phenotype, promotes epithelial tissue regeneration, and partially restores microbiota homeostasis.

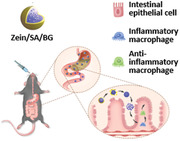

## 1 Introduction

Inflammatory bowel disease (IBD) is an idiopathic inflammatory disease of the gastrointestinal tract, characterized by chronic and recurrent inflammation of the gastrointestinal tract complicated with fibrosis in the intestine.^[^ ^1^ ^]^ IBD reduces the quality of patients’ life, increases the risk of intestinal cancer and even leads to death. Unfortunately, IBD has a high incidence, affecting over 3.5 million people worldwide.^[^ ^2^ ^]^ The specific pathogenesis of IBD has not been fully revealed. Current researches have suggested that IBD is linked to genetic, environmental, and microbial factors.^[^ ^3^, ^4^, ^5^ ^]^ Drugs including corticosteroids, aminosalicylates, antibiotics, and immunosuppressants are traditionally used in the treatment of IBD.^[^ ^6^ ^]^ Given that IBD patients may experience relapse of the disease, the treatment often follows a step‐up model whereby the patients start with anti‐inflammatory agents (corticosteroids or immunosuppressants) and step‐up to biologics such as monoclonal anti‐tumor necrosis factor (anti‐TNF) antibodies if the initial drugs fail to control the disease.^[^ ^7^ ^]^ Compared with traditional drugs, these biologics have shown good mucosal remission and therapeutic effects with fewer systemic side effects.^[^ ^8^ ^]^ However, according to previous reports, around 30% of the patients suffering from IBD do not respond to TNF antagonists, and 30–40% of patients experience decreased efficacy after repeated dosing.^[^ ^9^ ^]^ Therefore, it is necessary to continuously explore and develop new therapeutic approaches for the treatment of IBD.

Although the pathogenesis of IBD has not been fully elucidated, it has been widely recognized that IBD is associated with chronic inflammation in the intestine. Among different types of immune cells contributing to inflammation, macrophages that reside in the subepithelial lamina propria of intestine represent the most abundant mononuclear phagocytes in the body and are heavily involved in the maintenance of intestinal homeostasis.^[^ ^10^ ^]^ Intestinal macrophages constitute the first‐line defense by phagocytosing foreign substances, microbes, and dead cells. They can also interact with microbes and host cells by secreting multiple cytokines, such as IL‐1 and TNF‐alpha.^[^ ^11^ ^]^

Most of the macrophages resident in normal intestine tissue are anti‐inflammatory M2 macrophages.^[^ ^12^ ^]^ In the case of IBD, large number of macrophages, most of which are pro‐inflammatory M1 macrophages, infiltrate the mucosa, which can lead to disruption of the intestinal barrier.^[^ ^13^ ^]^ The inflammatory M1 macrophages secrete a large amount of pro‐inflammatory cytokines, including TNF‐alpha and IL‐1, which damage the intestine by causing epithelial cell apoptosis, destroying the barrier function, and resulting in the formation of granuloma and fibrosis.^[^ ^14^ ^]^ For example, studies have shown that TNF‐alpha at a high concentration can cause excessive inflammation and tissue damage.^[^ ^15^ ^]^

Our previous experiments have demonstrated that BG can modulate the behavior and polarization of macrophages, thereby enhancing tissue regeneration.^[^ ^16^ ^]^ BG was first developed as a bone regeneration material in 1969,^[^ ^17^ ^]^ and has been widely applied to repair a variety of bone defects in clinical surgery. BG can affect cellular activities during tissue regeneration via its dissolved ions, which can trigger various biological activities including osteogenesis, angiogenesis, and anti‐inflammation.^[^ ^18^, ^19^ ^]^ Previous research has suggested that BG can reduce the inflammatory response by modulating the activity of local immune cells to establish a suitable microenvironment for osteogenic differentiation of stem cells.^[^ ^20^ ^]^ Besides, BG can modulate macrophages by presenting physical, chemical, and biological signals.^[^ ^21^, ^22^ ^]^ Consequently, BG have been widely used clinically with relatively low cytotoxicity and few side effects.^[^ ^23^, ^24^ ^]^ Given the anti‐inflammatory properties of BG, we hypothesized that BG can be applied to treat IBD via modulating the polarization of intestinal macrophages.

Up to date, there was no report on applying BG for IBD treatment. One challenge that needs to be overcome is the development of an optimal delivery strategy. While the oral route can ensure convenience, high patient compliance, and direct delivery to the intestinal mucosa, oral delivery of BG is potentially hampered with premature BG dissolution and absorption since BG can react with acidic gastric juice quickly to release its ions in the stomach, thus limiting the amount of drug reaching the intestine.^[^ ^17^ ^]^ In view of the absence of an effective oral delivery vehicle for BG, we developed core–shell hydrogel microspheres consisting of sodium alginate (SA) as core and zein protein as shell to protect BG from the acidic environment in the stomach and release BG in the intestine. Zein is the primary storage protein of the corn,^[^ ^25^ ^]^ and can form a tough, glossy, hydrophobic and oil‐resistant coating for delivering food and drugs. Zein protein shell can protect the drug payload from the gastric acid, while it is readily degraded by pancreatic enzymes for drug release in the intestine.^[^ ^26^, ^27^, ^28^, ^29^ ^]^ It is also relatively safe and has been approved by the US Food and Drug Administration (FDA) as generally recognized as safe (GRAS). Meanwhile, SA has also been approved by FDA for human uses and utilized to generate microspheres for drug delivery. While the rapid dissolution of BG can produce a high pH that can cause tissue damage, the combination of SA and BG was shown to produce hydrogel microspheres that delay the dissolution of BG and result in a less alkaline environment according to our previous studies.^[^ ^16^, ^30^ ^]^ The development of therapeutic agent using FDA‐approved materials (BG, SA, and zein) shall accelerate the clinical translation of the therapeutic strategy.

**Figure 1:**
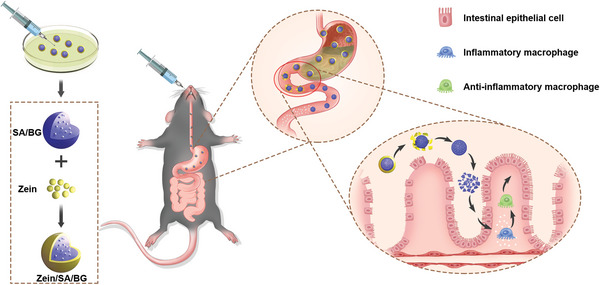
Schematic diagram of the preparation of Zein/SA/BG core–shell hydrogel microspheres and oral delivery of the microspheres for IBD treatment.

In this study, we first cultured macrophages (RAW264.7 (RAW)) in culture medium containing BG ions and LPS to assess the anti‐inflammatory effect of BG. Macrophages can be induced to a pro‐inflammatory state by exposed to cytokines secreted from Th1 lymphocytes or microbial products, such as LPS. LPS can bind to the Toll‐like receptor 4, which then triggers signaling cascades (e.g., NFkB and MAPK) and leads to the production of proinflammatory cytokines (e.g., TNF*α*, IL‐1*β*, IL‐6).^[^ ^31^ ^]^ We later cultured primary intestinal organoids using the conditioned medium collected from macrophage culture to further determine the effect of anti‐inflammation on an in vitro intestinal model. Next, we developed Zein/SA/BG hydrogel microspheres. Briefly, Zein/SA/BG microspheres are characterized by a core–shell structure, in which the core is composed of SA mixed with BG powders, and the shell consists of zein protein. The zein protein coating/shell can protect the core from the acidic environment in the stomach. Once the microspheres reach the intestine, the zein protein and alginate will be digested enzymatically to release BG to exert therapeutic effect. To show that the microsphere design allows targeted release of BG in the intestine, the degradation of Zein/SA/BG microspheres in simulated gastric fluids (SGF) and simulated intestinal fluids (SIF) was assessed. Finally, using both dextran sulfate sodium (DSS)‐induced acute and chronic colitis model, we evaluated the effect of Zein/SA/BG on reducing inflammation, promoting epithelial tissue regeneration, and regulating the microbiota balance in the intestine (**Figure** **1**). Overall, we demonstrate for the first time the previously unrecognized effect of BG in treating IBD via modulating macrophage polarization and promoting tissue regeneration. Our findings might provide a new strategy for the treatment of IBD.

## 2 Result

### 2.1 The Beneficial Effects of BG on Inflammatory Macrophages and Intestinal Organoids

**Figure 2:**
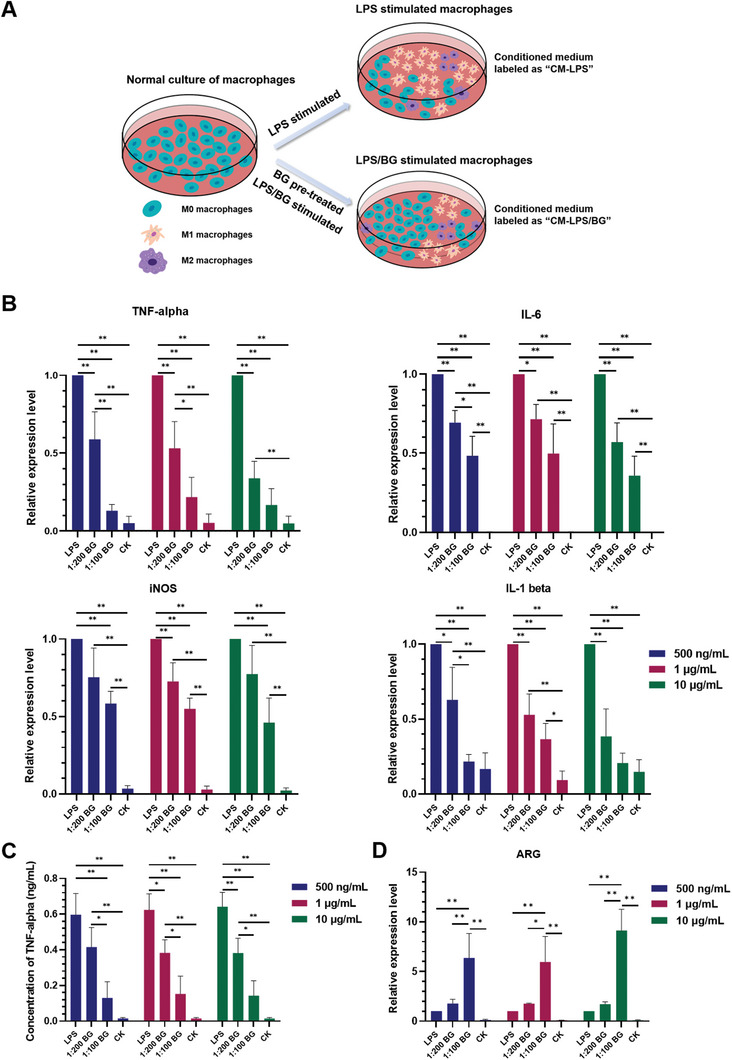
Effect of BG on inflammatory macrophages stimulated with different concentrations of LPS (500 ng mL^‐1^, 1 µg mL^‐1^, 10 µg mL^‐1^). A) Schematic diagram of various treatments of RAW and the collection of conditioned media. B) Expression of pro‐inflammatory genes (*TNF‐alpha, IL‐6, iNOS, IL‐1 beta*) in RAW after different treatments. (“LPS”: stands for incubation of RAW with LPS; “BG”: stands for incubation of RAW with LPS following pretreatment with BG extract liquid at 1:100 or 1:200 dilution ratio; “CK”: stands for RAW cultured with normal medium) C) Concentration of TNF‐alpha in the culture media in RAW after different treatments. D) Expression of anti‐inflammatory gene *ARG* in RAW after different treatments. (*n* = 3, * indicates *p* < 0.05, ** indicates *p* < 0.01)

IBD is characterized by chronic inflammation in the intestines and destruction of the intestinal epithelial barrier. To determine the role of BG in modulating the inflammatory response, RAW were activated with LPS in the presence or absence of BG extract liquids (**Figure** **2**A). The BG extract liquids have been demonstrated to contain dissolved ions of BG according to our previous study.^[^ ^32^ ^]^ By screening a range of LPS concentrations (500 ng mL^‐1^, 1 µg mL^‐1^, 10 µg mL^‐1^) reported to activate M1 polarization of RAW in literature,^[^ ^33^ ^]^ we showed that exposure to all concentrations tested resulted in upregulation of pro‐inflammatory genes, including *TNF‐alpha, iNOS, IL‐6, and IL‐1 beta*, compared with the non‐treated control (CK) (Figure 2B). Notably, the pre‐treatment of BG extract liquids prior to LPS exposure led to a reduction of pro‐inflammatory effects, the extent of which depended on the dilution ratio of BG extract liquids in RAW culture media. Specifically, the dilution ratio of 1:100 appeared to be more effective than 1:200 in reducing the level of pro‐inflammatory gene expression. Since TNF‐alpha is an important cytokine involved in IBD and other inflammatory diseases, ELISA was performed on the conditioned media collected from RAW culture to determine the level of TNF‐alpha production. Similar to the results of gene expression analysis, the concentration of TNF‐alpha increased following the exposure to LPS, but the increase was attenuated when RAW were pretreated with BG extract liquid (Figure 2C). Meanwhile, the expression of anti‐inflammatory gene *ARG* was significantly enhanced following the treatment of BG extract liquids (Figure 2D). Again, BG extract liquids diluted at 1:100 outperformed that at 1:200. In the following experiments, the dilution ratio of 1:100 was used.

The rescue and regeneration of damaged intestinal epithelium is critical to IBD treatment. To evaluate the role of BG on the damaging inflammatory effect to intestinal epithelium, conditioned media were collected from untreated RAW “CM‐RAW‐CK,”LPS‐activated RAW “CM‐LPS”, and LPS‐activated RAW after pretreatment of BG extract liquids (1:100 dilution ratio) “CM‐LPS/BG.” Since conventional intestinal cell lines are derived from tumor and may not be representative of native epithelium, we cultured intestinal epithelial organoids from isolated intestinal crypts of primary tissues following established protocol.^[^ ^34^ ^]^

**Figure 3:**
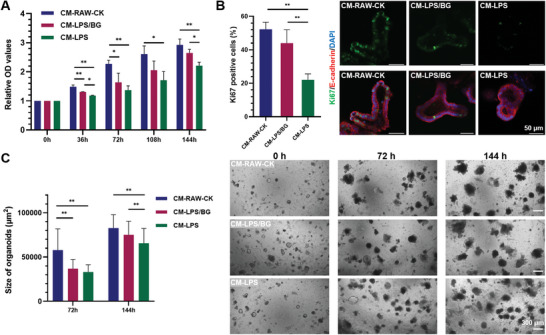
Influence of different conditioned media on the viability, proliferation and growth of intestinal organoids. A) CCK‐8 test of intestinal organoids cultured with different conditioned media. B) Immunofluorescence staining of Ki67 in intestinal organoids cultured with different conditioned media for 144 h. C) Morphology and size of intestinal organoids cultured with different conditioned media for 72 and 144 h. (*n* = 3, * indicates *p* < 0.05, ** indicates *p* < 0.01)

After intestinal organoids were treated with different conditioned media that were mixed with fresh organoid media at a ratio of 1:1, a decrease in cell viability was observed following CM‐LPS/BG and CM‐LPS treatments. However, the reduction was less significant after CM‐LPS/BG treatment (**Figure** **3**A). In addition, Ki67 staining of the organoids revealed that the ratio of Ki67 positive signals declined following CM‐LPS and CM‐LPS/BG treatments compared with CM‐RAW‐CK, but significantly more Ki67 positive signals were observed in the intestinal organoids cultured with CM‐LPS/BG than CM‐LPS (Figure 3B), which is consistent with the result of CCK‐8 assay. Moreover, by quantifying the organoid sizes at different time points (72 h and 144 h), the same trend was observed, where both CM‐LPS and CM‐LPS/BG treatments reduced organoid growth, but the intestinal organoids were significantly larger when cultured with CM‐LPS/BG than CM‐LPS (Figure 3C). These results showed that the conditioned media of inflammatory RAW (stimulated by LPS solution) contained cytokines, including TNF‐alpha that is widely regarded to be involved in IBD, which could injure the intestinal organoids. However, the pretreatment of BG extract liquids reduced the production of inflammatory cytokines, which then mitigated the damage to the intestinal organoids. These results showed the protective role of BG in reducing inflammatory damage to the intestine and supported the further investigation of applying BG for treating IBD.

### 2.2 Characterizations of Zein/SA/BG Hydrogel Microspheres

**Figure 4:**
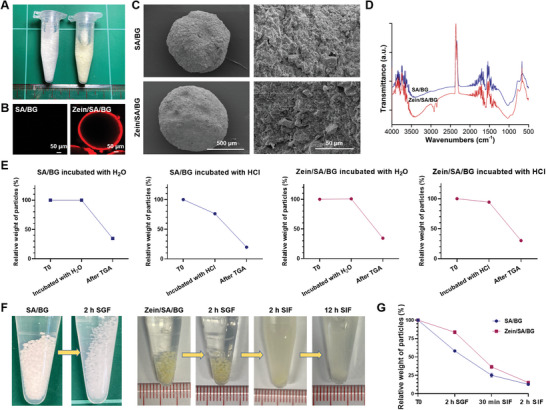
Characterization of the Zein/SA/BG and SA/BG hydrogel microspheres. A) Gross images of the Zein/SA/BG and SA/BG microspheres. B) Nile blue staining of the Zein/SA/BG and SA/BG microspheres. C) SEM images of the frozen dried SA/BG and Zein/SA/BG microspheres. D) FTIR results of the SA/BG and Zein/SA/BG microspheres. E) Weight changes of the SA/BG and Zein/SA/BG microspheres after incubation with H_2_O or HCl and after TGA test. F) Morphological changes of the SA/BG microspheres and Zein/SA/BG microspheres after incubated with SGF or SIF. G) Relative weights of the SA/BG and Zein/SA/BG microspheres after incubated with SGF and SIF.

While oral intake of BG can facilitate direct drug delivery to the intestine, the approach is limited by the fact that BG can react with acidic gastric acid to quickly dissolve in the stomach. To circumvent the problem, Zein/SA/BG hydrogel microspheres were fabricated. SA/BG hydrogel microspheres were also fabricated as control. **Figure** **4**A shows the morphology of the SA/BG and Zein/SA/BG microspheres that were 502 ± 16 µm and 650 ± 32 µm in diameter, respectively. The SA/BG microspheres appeared white while the Zein/SA/BG microspheres appeared yellowish. Except for the color, no distinct differences between these two types of microspheres were observed. After the microspheres were stained with Nile blue, a red fluorescence layer was observed on the surface of Zein/SA/BG microspheres, indicating the presence of protein (zein) (Figure 4B). To evaluate the influence of the zein protein coating on the morphology of the microspheres, SEM was performed to record the surface morphology of the microspheres. No distinct difference can be observed between the surface of frozen‐dried SA/BG and Zein/SA/BG microspheres (Figure 4C), suggesting that the zein coating did not notably affect the surface morphology of the microspheres. FTIR analysis on the microspheres further verified that the microspheres contained zein protein, as demonstrated by the presence of two distinctive peaks between 2800 and 3000 cm^−1^, which is consistent with the results reported in literature (Figure 4D).^[^ ^35^ ^]^ Finally, cytotoxicity assay showed that Zein/SA/BG microspheres did not compromise cell viability (Figure S1, Supporting Information), proving that the microspheres are biocompatible.

### 2.3 Targeted Release of BG from Zein/SA/BG Hydrogel Microspheres in Simulated Intestinal Environment

Next, the protective effects of the core–shell structure of Zein/SA/BG in the gastric environment were evaluated. After the SA/BG and Zein/SA/BG hydrogel microspheres were immersed in dd H_2_O for 24 h, there was little change in the microsphere weight. Afterward, the microspheres were analyzed with thermogravimetric analysis (TGA) and the results showed that similar percentage of remaining weight (≈34%) after heating to 900 °C (which was above the melting temperature of zein and SA) was detected for two types of microspheres (Figure 4E). When the freshly made microspheres were soaked in HCl for 24 h, SA/BG microspheres showed a more drastic decrease of weight (≈24%) than Zein/SA/BG microspheres (≈5%). Subsequently, after the microspheres were analyzed with TGA again, the percentages of remaining weight of SA/BG and Zein/SA/BG microspheres were ≈20% and ≈30%, respectively (Figure 4E). Given that the original weight of BG in all microspheres was the same, the difference of the remaining weight after TGA (which was contributed mainly by the presence of BG) between the two types of microspheres was likely due to the different amount of BG loss during HCl incubation. Our results suggested that more BG was lost from the SA/BG microspheres than from the Zein/SA/BG microspheres following HCl incubation. We also noticed that the weight of the Zein/SA/BG microspheres remaining after HCl incubation and TGA test was similar to that after dd H_2_O incubation and TGA test (30%‐34%), indicating that the HCl incubation did not cause significant amount of BG from the Zein/SA/BG microspheres to dissolve. Therefore, the zein protein coating effectively protected BG from being released in the acidic environment.

The two types of hydrogel microspheres were next incubated with SGF to evaluate the effects of the simulated gastric environment on the microspheres (Figure 4F). After incubated with the SGF for 2 h to simulate the gastric retention time for food, SA/BG microspheres became smaller and transparent, while there was no distinct change in terms of size and color of the Zein/SA/BG microspheres, further proving the stability of the Zein/SA/BG microspheres in gastric environment. When the Zein/SA/BG microspheres were further incubated with SIF for 2 h, the microspheres quickly dissolved and, after 12 h that represent the minimal time it takes for food to transit through the colon, all microspheres were invisible. During SGF incubation, the Zein/SA/BG microspheres exhibited reduced weight loss compared with the SA/BG microspheres (Figure 4G). During SIF incubation, both types of microspheres showed rapid weight loss indicating the dissolution of the microspheres. All these results suggested that the Zein/SA/BG microspheres could protect BG from premature release in the gastric environment and achieve targeted release of BG in the intestinal environment.

### 2.4 In Vivo Evaluation of Therapeutic Potential of Zein/SA/BG Microspheres in Acute Colitis Mice

**Figure 5:**
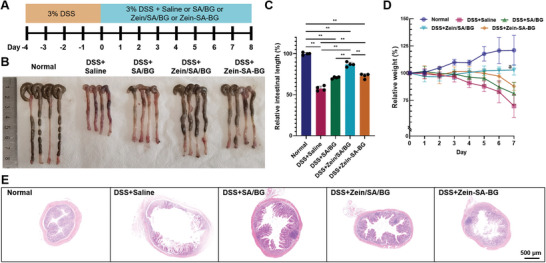
Therapeutic effect of BG‐based treatment in DSS‐induced acute colitis mice. A) Timeline of the in vivo experiment. B) Gross image of the large intestine from normal mice and DSS‐induced mice after different treatments. (“Normal” stands for the large intestine from normal mice; “DSS+Saline” stands for the large intestine from DSS‐induced mice treated with saline; “DSS+SA/BG” stands for the large intestine from DSS‐induced mice treated with the SA/BG microspheres; “DSS+Zein/SA/BG” stands for the large intestine from DSS‐induced mice treated with the Zein/SA/BG microspheres; “DSS+Zein‐SA‐BG” stands for the large intestine from DSS‐induced mice treated with the mixture of Zein protein, SA and BG powders) C) Relative length of large intestine of normal mice and DSS‐induced mice after different treatments. D) Relative weight changes of normal mice and DSS‐induced mice after different treatments. E) H&E staining of the large intestine from normal mice and DSS‐induced mice after different treatments. (*n* = 4, ** indicates *p* < 0.01, “a” indicates *p* < 0.05 between DSS+Zein/SA/BG and normal and *p* < 0.01 between DSS+Zein/SA/BG and {DSS+Zein‐SA‐BG, DSS+SA/BG, and DSS+saline}).

After demonstrating that BG inhibited inflammatory response and damage to intestinal organoids in vitro and that Zein/SA/BG hydrogel microspheres were able to prevent premature release of BG, we evaluated the therapeutic potential of Zein/SA/BG microspheres for IBD treatment in vivo. As shown in **Figure** **5**A, an acute colitis mice model was established by feeding mice with DSS to induce acute inflammation. Afterward, Zein/SA/BG microspheres, SA/BG microspheres and a mixture of zein, SA and BG (Zein‐SA‐BG) were administered to the mice daily. After 7 d, the mice were sacrificed, and the large intestinal tissues were collected and imaged (Figure 5B). A significant reduction of intestinal length was observed following DSS+saline treatment. While all Zein/SA/BG, SA/BG and Zein‐SA‐BG treatments led to partial recovery of intestinal lengths, the effect of Zein/SA/BG was the most significant (Figure 5C). Similarly, there was a reduction of mice weight after DSS+saline treatment (Figure 5D). Treatments with Zein/SA/BG, SA/BG and Zein‐SA‐BG all attenuated the weight decrease and the effect was the most significant in the Zein/SA/BG group. In addition, H&E staining showed that DSS treatment led to tissue swelling and destruction of crypt structures in the mucosa layer (Figure 5E), but the damage was partially reversed after treatments with Zein/SA/BG, SA/BG and Zein‐SA‐BG. When comparing the H&E images of SA/BG, Zein/SA/BG and Zein‐SA‐BG groups, it appeared that the lowest degree of tissue swelling was observed in Zein/SA/BG group. All these results suggested that Zein/SA/BG microspheres outperformed SA/BG and Zein‐SA‐BG and could be used to treat the DSS‐induced acute injury. Using the acute colitis model, we also compared the therapeutic efficacy of Zein/SA/BG microspheres with that of 5‐aminosalicylic acid (5‐ASA), the drug typically used to treat IBD clinically. Our results indicated that their efficacies are comparable based on analyses of intestinal length, weight of mice, and intestinal tissue morphology (Figure S2, Supporting Information).

**Figure 6:**
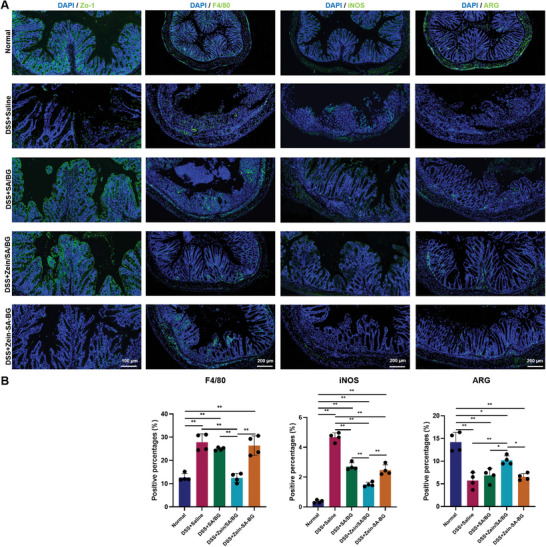
Characterization of intestinal tissue of normal mice and DSS‐induced acute colitis mice after treatment. A) Immunostaining of ZO‐1, F4/80, iNOS and ARG in the large intestine of normal mice and DSS‐induced acute colitis mice after different treatments. B) Quantification of expression of ZO‐1, F4/80, iNOS and ARG in the samples. (*n* = 4, * indicates *p* < 0.05, ** indicates *p* < 0.01).

Next, immunostaining of ZO‐1, a tight junction marker, was performed to evaluate the regeneration of the intestine epithelium. The loss of tight junction is closely related to the increase of intestinal permeability in IBD patients. To restore the intestinal barrier, the tight junction needs to be reconstructed. **Figure** **6**A demonstrated that ZO‐1 signals were lost in DSS‐induced mice treated with saline (DSS+saline) compared with normal mice, suggesting that DSS treatment destroyed the intestinal barrier. When SA/BG microspheres and Zein/SA/BG microspheres were administered, the impaired intestine epithelium recovered by expressing ZO‐1 in the intestine epithelium. To evaluate the inflammatory condition of the large intestine, F4/80 staining was performed to label the macrophages in the intestinal tissue and the positive staining was quantified (Figure 6A,B). Our results showed that significantly more F4/80 positive signals were observed in the large intestinal tissues of DSS‐induced mice treated with saline (DSS+saline), SA/BG microspheres (DSS+SA/BG) and Zein‐SA‐BG mixture (DSS+Zein‐SA‐BG) compared with normal mice (Normal) and DSS‐induced mice treated with Zein/SA/BG microspheres (DSS+Zein/SA/BG). Since the macrophages can by categorized into subtypes such as M1 and M2 macrophages, we performed further analysis to determine their identity. Immunostaining of iNOS, CD86 and CD80, which are markers of M1 macrophages, was performed on the intestinal tissues (Figure 6 and Figure S3, Supporting Information). In general, increased expression of iNOS, CD86 and CD80 was observed after DSS treatment, indicating more M1 macrophages were present during inflammation. A significant reduction of M1 macrophage markers was observed following the administration of Zein/SA/BG microspheres. Meanwhile, immunostaining of ARG, a marker of M2 macrophages, was performed on the large intestinal tissues to determine the presence of M2 macrophages that are anti‐inflammatory (Figure 6A). Quantification of ARG signals shows that there were significantly fewer ARG positive cells in the mucosa tissue of all DSS‐induced mice (Figure 6B). This demonstrated that DSS treatment led to a decrease of M2 macrophages in the large intestine. Comparing the four DSS treatment groups, DSS+Zein/SA/BG group exhibited significantly larger number of ARG positive cells than other groups, indicating that the Zein/SA/BG microspheres were more effective in enhancing the amount of M2 macrophages in the DSS‐induced mice. Taken together, our results showed that DSS treatment resulted in an increased number of macrophages, particularly M1 macrophages, and decreased number of M2 macrophages in the intestinal tissue. Since M1 macrophages produce a large amount of inflammatory cytokines, this might lead to the destruction of the intestinal barrier. Compared with SA/BG microspheres and Zein‐SA‐BG mixture, Zein/SA/BG microspheres were more effective in reducing inflammation and promoting the restoration of the barrier.

IBD is often associated with dysbiosis of gut microbiome, such as shifting of the composition of the intestinal microbiota. Research has suggested that certain microorganism alterations are associated with the immune reactions in the intestine and the pathogenesis of IBD.^[^ ^36^ ^]^ To examine the effect of DSS and BG treatment on intestinal microbiota, 16S ribosomal RNA (rRNA) sequencing was performed. Our results showed that the relative abundances of microbiota at the phylum level, order level, family level, and genus level were all altered following DSS treatment (Figure S4A, Supporting Information). Compared with DSS+saline, treatments of DSS+Zein/SA/BG and DSS+Zein‐SA‐BG resulted in partial restoration of the microbial balance. This was demonstrated in the comparative coordinate analysis (Figure S4B, Supporting Information). While all DSS‐induced groups were found distant from the normal control (Group A) in the plot, DSS+Zein/SA/BG and DSS+Zein‐SA‐BG groups were located closer to normal control than DSS+saline. Our results suggested that oral administration of Zein/SA/BG microspheres and Zein‐SA‐BG mixture could partially restore the balance of intestinal microbiota in the inflammatory intestine. Finally, the tissues of, liver, spleen, and kidney of normal mice and DSS‐induced mice after different treatments were collected and stained with H&E and the results are shown in Figure S5 (Supporting Information). No obvious difference could be observed among different groups, suggesting no serious side effects were observed following the oral delivery of Zein/SA/BG microspheres for one week. In summary, treatment with Zein/SA/BG could reduce inflammation in the intestine, enhance the regeneration of intestinal tissue, and partially restore the microbiota balance in the acute colitis model.

### 2.5 In Vivo Evaluation of Therapeutic Potential of Zein/SA/BG Microspheres in Chronic Colitis Mice

**Figure 7:**
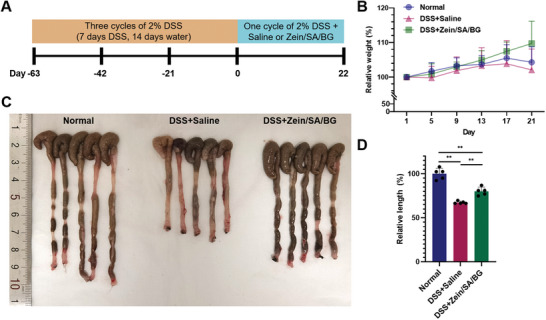
Therapeutic effect of BG‐based treatment in DSS‐induced chronic colitis mice. A) Timeline of the in vivo experiment. B) Weight changes of normal mice and chronic colitis mice after different treatments. (“Normal” stands for the normal mice; “DSS+Saline” stands for the chronic colitis mice treated with saline; “DSS+Zein/SA/BG” stands for the chronic colitis mice treated with Zein/SA/BG microspheres). C) Gross image of the large intestines from normal mice and chronic colitis mice after different treatments. D) Relative length of large intestine of normal mice and chronic colitis mice after different treatments. (*n* = 5, ** indicates *p* < 0.01)

While the acute IBD model allows us to analyze short‐term alteration of intestinal barrier and immune responses, chronic IBD model will better recapitulate the chronic nature of IBD and facilitate investigation into the long‐term complications such as tissue fibrosis.^[^ ^37^ ^]^ Here, chronic colitis mice were induced by administering three cycles of 2% DSS solution to the mice for a total of 63 days based on a reported protocol (**Figure** **7**A).^[^ ^37^ ^]^ The mice were then given Zein/SA/BG microspheres as treatment and saline as control. The weight and large intestinal length of the normal mice (Normal), DSS‐induced mice with chronic colitis treated with Zein/SA/BG microspheres (DSS+Zein/SA/BG), and DSS‐induced mice with chronic colitis treated with saline (DSS+saline) were recorded and normalized to the weight before treatment and the intestinal length of the normal mice, respectively (Figure 7B,D). Our results showed that repeated administration of DSS resulted in a reduction in mice weight and intestinal length. Compared with DSS+saline, the administration of Zein/SA/BG microspheres led to increased weight and intestinal length, suggesting that these microspheres exhibited therapeutic effect in reducing the symptoms of the chronic colitis.

**Figure 8:**
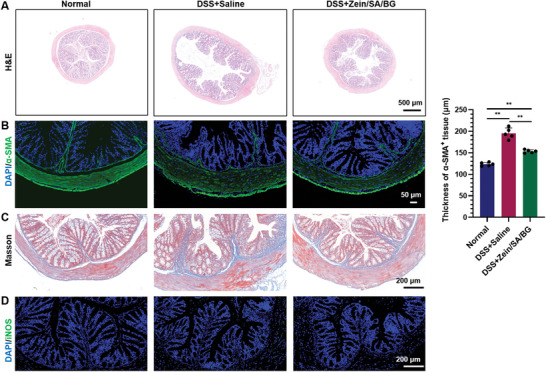
Characterization of the intestinal tissue of normal mice and DSS‐induced chronic colitis mice after treatment. A) H&E staining of the large intestine of normal mice and chronic colitis mice treated with saline and Zein/SA/BG microspheres. B) Immunostaining of *α*‐SMA in large intestine of normal mice and chronic colitis mice treated with saline and Zein/SA/BG microspheres. C) Masson staining of large intestine of normal mice and chronic colitis mice treated with saline and Zein/SA/BG microspheres. D) Immunostaining of iNOS in large intestine of normal mice and chronic colitis mice treated with saline and Zein/SA/BG microspheres. (*n* = 5, ** indicates *p* < 0.01).

H&E staining was then performed on the intestinal samples collected from the three groups (**Figure** **8**A). Compared with normal mice, DSS+saline and DSS+Zein/SA/BG groups both displayed altered crypt structures, indicating damage caused by chronic exposure to DDS. Besides, the diameter of large intestine of chronic colitis mice was larger than that of normal mice, suggesting that the chronic colitis led to tissue swelling. Between DSS+saline and DSS+Zein/SA/BG, more complete recovery of crypt structure was observed in the DSS+Zein/SA/BG group. Moreover, the degree of tissue swelling in Zein/SA/BG was lower in the DSS+Zein/SA/BG group than the DSS+saline group. These results further proved that oral delivery of Zein/SA/BG microspheres contributed to the regeneration of the intestine in chronic colitis mice.

Fibrosis is a common feature in the chronic colitis and is characterized by excessive deposition of extracellular matrix, such as collagens, in the intestine.^[^ ^38^, ^39^ ^]^ A variety of cell types are involved in the development of fibrosis, including intestinal subepithelial myofibroblasts and smooth muscle cells which express *α*‐SMA.^[^ ^40^ ^]^ To investigate the influence of Zein/SA/BG microspheres on the fibrotic condition, immunostaining of *α*‐SMA was performed (Figure 8B). Our results showed that both DSS+saline and DSS+Zein/SA/BG groups contained thicker *α*‐SMA positive tissue layer than the normal mice. Between the two groups, DSS+Zein/SA/BG group showed reduced thickness in the *α*‐SMA positive tissue layer, suggesting that Zein/SA/BG microspheres reduced the presence of cell types that contributed to fibrosis. Masson staining for collagen showed that although more collagen (labeled as blue) was present in the submucosa and muscle layer of both DSS+saline and DSS+Zein/SA/BG groups compared with the normal control (Figure 8C), DSS+Zein/SA/BG group displayed less blue fibers than DSS+saline group. This confirmed the therapeutic effect of Zein/SA/BG microspheres to inhibit fibrosis. Finally, immunostaining of iNOS was performed to determine the presence of M1 macrophages in the intestinal tissues (Figure 8D). The results showed that less iNOS positive signals were observed in the chronic colitis mice following the treatment with Zein/SA/BG than saline and demonstrated that Zein/SA/BG microspheres reduced inflammation in the large intestine of chronic colitis mice.

**Figure 9:**
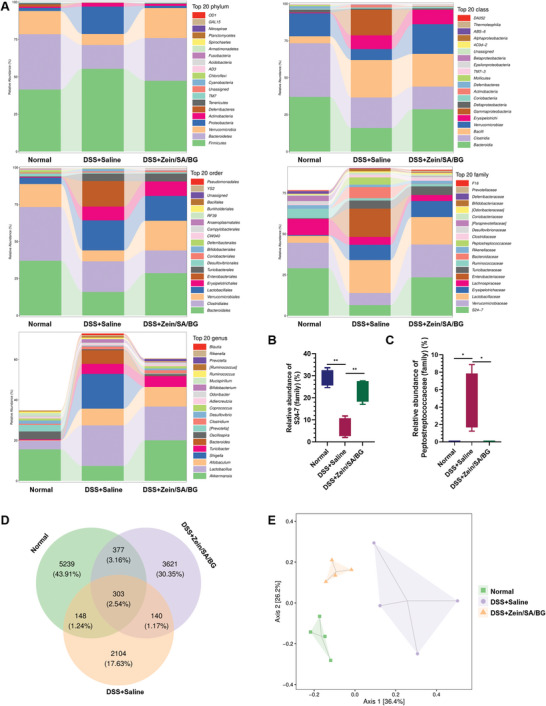
Effect of Zein/SA/BG microspheres on intestinal microbiota in chronic colitis mice. A) Distribution of intestinal microbiota species of normal mice, DSS‐induced chronic colitis mice treated with saline and Zein/SA/BG microspheres at the phylum level, class level, order level, family level and genus level. B) Relative abundance of S24‐7 (family). C) Relative abundance of peptostreptococcaceae (family). D) Venn diagram of common and unique intestinal microbiota species from normal mice and DSS‐induced chronic colitis mice after different treatments. E) Principal coordinate analysis (PCoA) of intestinal microbiota from normal mice and DSS‐induced chronic colitis mice after different treatments.

Furthermore, 16S rRNA sequencing was performed to examine the effect of Zein/SA/BG on the intestinal microbiota in the chronic colitis mice. **Figure** **9**A displays the relative abundances of intestinal microbiota at the phylum level, class level, order level, family level, and genus level. From the results, it can be concluded that the chronic colitis led to microbiota imbalance, but the balance was partially restored following the treatment with Zein/SA/BG. In particular, the relative abundance of S24‐7, which is related to carbohydrate fermentation and barrier function repair in inflamed intestine,^[^ ^41^, ^42^ ^]^ decreased significantly in DSS+saline group compared with DSS+Zein/SA/BG group and normal control, while its relative abundance was not significantly different between DSS+Zein/SA/BG group and normal control (Figure 9B). Besides, the relative abundance of peptostreptococcaceae, which is enhanced in the inflamed intestine and is associated with multiple host genes and pathways in IBD,^[^ ^43^ ^]^ was significantly higher in DSS+saline group than DSS+Zein/SA/BG group and normal control, while its relative abundance was not significantly different between DSS+Zein/SA/BG group and normal control (Figure 9C). This suggested that Zein/SA/BG might mitigate chronic IBD via promoting the growth of microbiota that participate in intestinal repair while inhibiting those that are involved in the IBD pathogenesis. Meanwhile, the Venn diagram of the common and unique intestinal microbiota species showed that DSS+saline group (2695) contained fewer species than the DSS+Zein/SA/BG (4441) and normal control (6067) groups (Figure 9D), which suggested that chronic inflammation reduced the diversity of gut microbiome and it was partially rescued following Zein/SA/BG treatment. Besides, compared with the DSS+saline group, more common species were identified between DSS+Zein/SA/BG group and normal control than between DSS+saline group and normal control. The comparative cylindrical coordinate analysis suggested that the DSS+Zein/SA/BG group was located closer to the normal group than the DSS+saline group (Figure 9E). In short, Zein/SA/BG treatment partially restored the microbiota balance seen in normal control that may contribute to reduced inflammation and enhanced tissue regeneration.

Finally, the tissues of liver, spleen, and kidney of the three groups were collected and stained with H&E and the results are shown in Figure S6 (Supporting Information). Our results showed that the tissue structure of spleen appeared distorted, which might be a side effect of long‐term DSS treatment. No obvious difference could be observed between Zein/SA/BG group and normal control, suggesting no serious side effects were observed following the oral delivery of Zein/SA/BG microspheres.

## 3 Discussion

Inflammatory bowel disease is a chronic, systemic and relapsing immune‐mediated inflammatory disease with significant morbidity, leading to poor quality of patients’ life.^[^ ^44^ ^]^ The incidence and prevalence of IBD are increasing worldwide, especially in China.^[^ ^45^ ^]^ Given the limitations of current therapeutic treatments, an alternative treatment option is needed. In the present study, using in vitro experiments, we revealed that BG reduced inflammatory response of macrophages and subsequently relieved the damage to intestinal organoids. Our proposed core–shell configuration of Zein/SA/BG protected BG from the acidic environment and released it in the intestinal environment. Using in vivo acute and chronic IBD models, we demonstrated that Zein/SA/BG reduced tissue inflammation, promoted regeneration of intestinal tissue, and partially restored microbiota homeostasis. Our results supported further development of Zein/SA/BG microspheres as potential IBD treatment.

To date, environmental factors, genetic defects, microbiota imbalance, and immune response have been identified as the major etiology of IBD. A series of pathological alterations, including disrupted intestinal barrier function, abnormal immune response, and unbalanced gut microbiome, contribute to the pathogenesis of IBD. Nevertheless, current therapeutic agents such as aminosalicylates and biologics (e.g., anti‐TNF) often fail to fully address the alterations and produce long‐term remission of IBD. Therefore, considerable attention has focused on the development of new therapeutic approaches. Among various intestinal cell types, intestinal macrophages are essential for establishing and maintaining intestinal homeostasis. The disorders and dysregulation of the intestinal macrophages are believed to result in the loss of the tolerance to intestinal bacteria and antigens from food, which are speculated to underlie the chronic inflammation of IBD and potentially serve as a promising therapeutic target.^[^ ^46^ ^]^

In our previous studies, BG was applied to modulate the macrophages in the skin wound towards M2 phenotype to enhance tissue regeneration.^[^ ^47^ ^]^ Here our results showed that BG attenuated the inflammatory damage to intestinal organoids in vitro, in part by reducing the secretion of TNF‐alpha, which is closely related to the pathogenesis of IBD, from inflammatory macrophages. Compared with 2D culture of epithelial cell lines, intestinal organoids can better simulate the intestinal epithelial tissues and therefore were used as a model to simulate the response following inflammation.^[^ ^48^ ^]^ Before evaluating the efficacy of BG in vivo, an optimal delivery strategy was needed. While oral delivery of BG facilitates drug uptake at the intestine, BG can react with gastric acid and dissolve in the stomach. To prevent the premature dissolution of BG and absorption of ions in the acidic gastric environment, we designed and fabricated Zein/SA/BG microspheres. We demonstrated that the Zein coating effectively prevented the digestion of the microspheres in the SGF, while both Zein and SA were digested in the SIF to release BG. By modulating the inflammatory environment, the Zein/SA/BG microspheres effectively reduced inflammation in the intestine, enhanced tissue regeneration, and partially restored microbiota balance in acute and chronic colitis models.

In this study, both acute and chronic IBD models were induced by administering DSS to mice. Although the inflammation caused by DSS is not exactly the same as the inflammation seen in the IBD patients, DSS‐induced mice are still the most commonly used inflammation model to investigate IBD.^[^ ^49^ ^]^ In our DSS‐induced mice model, destruction of the epithelial tissue barrier, tissue swelling, weight loss, and accumulation of M1 macrophages were observed, which is consistent with the clinical symptoms of IBD.^[^ ^49^ ^]^ Following treatment of Zein/SA/BG microspheres, reconstruction of the ZO‐1 protein layer in the epithelial tissue was observed. The ZO‐1 protein is essential for the reconstruction of the intestinal barrier, which depends on the integrity of the tight junction complexes between the epithelial cells.^[^ ^50^ ^]^

Besides, Zein/SA/BG microspheres also reduced the number of iNOS‐positive signals labeling inflammatory macrophages. Upon inflammatory stimulation, iNOS can lead to the release of nitric oxide (NO). High concentration of NO in the intestine is associated with the chronic inflammation in IBD,^[^ ^51^, ^52^ ^]^ resulting in the production of peroxynitrite that will damage the tissue and aggravate inflammation.^[^ ^53^ ^]^ Moreover, we observed that Zein/SA/BG microspheres treatment attenuated intestinal fibrosis in the chronic colitis mice model. Intestinal fibrosis is one of the most common complications of IBD which affects more than 30% of patients, and is usually defined as the excessive accumulation of scar tissue in the intestinal wall.^[^ ^54^ ^]^ Scar tissue formation is induced by the chronic inflammation that promotes the proliferation and collagen secretion of the fibroblasts.^[^ ^54^ ^]^ Specifically, TNF‐alpha and other inflammatory cytokines can enhance the proliferation of myofibroblasts and smooth muscle cells, which leads to excessive collagen deposition. In our chronic colitis model, administration of DSS thickened the *α*‐SMA positive tissue layer in the intestinal wall, which suggested that the chronic colitis stimulated by the DSS led to the accumulation of the *α*‐SMA positive myofibroblasts/smooth muscle cells, an early sign of fibrosis in the inflammatory intestine. Following Zein/SA/BG treatment in chronic colitis mice, the thickness of the *α*‐SMA positive tissue layer was decreased, which resulted in the mitigation of the early‐stage fibrosis. Last but not least, Zein/SA/BG treatment resulted in partial restoration of microbiota imbalance in acute and chronic colitis mice. In particular, Zein/SA/BG was shown to promote the growth of microbiota that participate in intestinal repair (e.g., S24‐7), while inhibiting those that are involved in the IBD pathogenesis (e.g., peptostreptococcaceae) in chronic colitis mice. This suggested that BG‐based treatment may function similarly to other potential microbiome‐based therapeutics, such as probiotics and fecal microbiota transplantation, in treating IBD via modulating gut microbiome. Whether Zein/SA/BG affected the microbiota directly or indirectly through mediating responses of macrophages or other cell types is currently unknown and requires further investigation.

Our results demonstrate that BG is a promising therapeutic option for IBD since it has long history of clinical use with proven safety. Similarly, zein protein and SA are also approved by FDA for oral use, which supports the further development of Zein/SA/BG microspheres for clinical use. To our best understanding, our study is the first attempt to apply BG for IBD treatment through oral delivery. Compared with other therapeutic agents such as monoclonal antibodies, bioactive ions released from BG can potentially be more effective due to their quicker diffusion and longer half‐life than proteins or small molecules.^[^ ^55^ ^]^ Our results already indicated comparable efficacy of Zein/SA/BG microspheres and the widely used IBD drug 5‐ASA (Figure S2, Supporting Information). With further optimization of formulation and dosage, Zein/SA/BG is expected to outperform existing treatment options. Besides, the cost of BG is significantly lower than that of biologics such as anti‐TNF. Most importantly, our targeted delivery approach can ensure that most BG will be released in the intestinal environment. The strategy can be adopted, with modification, to achieve oral delivery of BG to other organs such as stomach and duodenum for treating various gastrointestinal diseases. The technology can even be adapted, such as by using microfluidics to generate smaller microspheres, for intravenous delivery of BG, which can open up new opportunities for applying BG to treat various internal diseases. In this study, we also revealed the effect of BG on gut microbiome for the first time, which will pave the way for more research in utilizing BG to modulate gut microbiome to treat various diseases.

## 4 Conclusion

In summary, our study demonstrates that BG is a promising therapeutic candidate for IBD treatment. Based on in vitro cell culture experiments, we demonstrated the beneficial effect of BG on attenuating inflammation induced by macrophages and subsequent damage to intestinal organoids model. Next, to facilitate the delivery of BG to the intestinal tissue, we developed Zein/SA/BG hydrogel microspheres for targeted release of BG in the intestinal environment. Finally, administering Zein/SA/BG microspheres to DSS‐induced acute and chronic colitis model resulted in reduced intestinal inflammation, enhanced epithelial tissue regeneration, and partial restoration of microbiota balance. All these findings suggested that BG has the potential to be further investigated as a therapeutic drug for IBD. The combination of clinically‐approved biomaterials (BG, SA, and zein) for treating IBD can be a promising and translational strategy that advances IBD treatment.

## 5 Experimental Section

### Experimental Design

The objective of this study was to investigate the therapeutic potential of BG in the treatment of IBD. First, in vitro cell culture experiment was performed to assess the effect of BG on LPS‐induced macrophages and inflammatory damage to intestinal organoids. Next, a core–shell hydrogel microsphere system (Zein/SA/BG) was developed for oral delivery of BG and was characterized. Finally, Zein/SA/BG was applied to treat acute and chronic colitis mice induced by DSS treatment, after which intestinal tissue regeneration, the inflammatory status of the intestine, and microbiota composition were evaluated.

### Analysis of the Effect of BG on the Proinflammatory Macrophages

The effect of BG on the proinflammatory macrophages was investigated by culturing macrophage cell line RAW264.7 (RAW) with BG extract liquids. Briefly, 1 g of the BG powders (Kunshan Overseas Chinese Technology New Materials Co., Ltd.) was sterilized by UV radiation for 15 min before they were added into a 6 cm cell culture dish and incubated with 5 mL of DMEM/F12 medium in the cell incubator for 24 h. After the incubation, the supernatant was collected and filtered through a 0.22 µm filter to obtain BG extract liquids for further use.

RAW was cultured in DMEM/F12 containing 10% FBS and 1% antibiotic‐antimycotic. BG extract liquids were diluted with fresh RAW culture medium at the ratio of 1:100 and 1:200 before they were added to the culture medium of RAW (around 85% confluency) for 2 hours to pre‐treat the RAW. Then, the lipopolysaccharide (LPS) solution used to activate the RAW was diluted with the culture medium to final concentrations of 500 mg mL^‐1^, 1 µg mL^‐1^ and 10 µg mL^‐1^, respectively. Afterward, normal RAW and BG pre‐treated RAW were incubated with the different LPS solutions with or without the addition of BG extract liquid for 24 h. The culture media of RAW cultured with different medium were collected as “conditioned medium” and the concentration of the TNF‐alpha in the media was measured by ELISA (ab212073, Abcam) following the manufacturer's instructions. Finally, the cells were digested, collected and their RNA was extracted for RT‐PCR analysis. RNA was transcribed into cDNA using HiScript III RT Super Mix before 5 µL of the TBBR Premix Ex Taq, 1 µL of primer of pro‐inflammatory genes (*TNF‐alpha, iNOS, IL‐6, IL‐1 beta*) and anti‐inflammatory gene *ARG*, and 4 µL of cDNA were mixed for RT‐PCR analysis using the QS 7 system (Quant Studio 7 Flex real time PCR System). The primers sequences are listed in **Table** **1**.^[^ ^16^ ^]^ The results were normalized to the expression of GAPDH which serves as the housekeeping gene and the relative gene expression was quantified by the 2−^ΔΔCt^ method.

**Table 1:** Primers sequences for RT‐qPCR

Gene	Forward primer	Reverse primer
*ARG*	CTCCAAGCCAAAGTCCTTAGAG	AGGAGCTGTCATTAGGGACATC
*TNF‐alpha*	TGAGAAGTTCCCAAATGGCCTC	CTACAGGCTTGTCACTCGAATTTTG
*IL‐6*	TGATGGATGCTACCAAACTGG	TTCATGTACTCCAGGTAGCTATGG
*iNOS*	GTTCTCAGCCCAACAATACAAGA	GTGGACGGGTCGATGTCAC
*IL‐1 beta*	GAGGATACCACTCCCAACAGACC	GATCCACACTCTCCAGCTGCA

### Analysis of the Effects of the Conditioned Media on the Development of Intestinal Organoids

The intestinal organoids were developed from the isolated intestinal crypt cells of mice intestine following the protocols mentioned previously.^[^ ^34^ ^]^ The intestinal organoids were seeded into 24‐well plates in Matrigel. The conditioned media collected from RAW with LPS or LPS/BG (where the concentration of LPS used was 1 µg mL^‐1^) pretreatment were mixed with the culture medium of the intestinal organoids (at the ratio of 1:1) before added to the organoid culture. At various time points (36, 72, 108, and 144 h), the viability of intestinal organoids cultured with different media was measured by Cell Counting Kit‐8 (CCK‐8) kit according to the manufactures’ instructions. The images of the organoids were taken with a light microscope at 72 and 144 h and the size of the organoids was quantified. To assess the proliferation of organoids cultured with different media, Ki67 staining was conducted. To perform Ki67 imaging, the organoids were collected, fixed by 4% PFA solution for 1 h at room temperature, and permeabilized by Triton‐X before the organoids were blocked by goat serum. The organoids were then incubated with the anti‐Ki67 primary antibodies (ab16667, Abcam) at the concentration of 1:100 and anti‐E‐cadherin primary antibodies (ab40772, Abcam) at the concentration of 1:200 for 24 h at 4 °C. Subsequently, the organoids were washed by PBS for three times, incubated with the secondary antibodies for 24 h at 4 °C, and imaged by confocal microscopy. The images were taken and the ratio of the Ki67‐positive cells in the organoids was calculated to represent the proliferation of the intestinal organoids cultured with different media.

### Preparation of Zein/SA/BG Hydrogel Microspheres

2 g SA powders (A2033, Sigma‐Aldrich) were added into 100 mL sterilized deionized water to produce a 2% solution under mixing. The solution was sterilized by filtering through a 0.22 µm filter. The 2% SA solution was stored at 4 °C until further use. Zein solution (Z3625, Sigma‐Aldrich) was prepared by adding 2 g of sterilized zein protein powders and 5 g of sterilized calcium chloride into 100 mL of 70% (v/v) ethanol solution.

BG powders were first sterilized by UV radiation for 10 min. Afterward, 0.2 g of BG was mixed with 0.8 mL 2% SA solution by using three‐limb tubes. After mixing, the BG/SA solution was injected into 25 mL zein solution (2% w/v). The zein solution containing SA and BG was placed onto a magnetic stirrer and stirred at 200 rpm for 1 h. Once the SA/BG liquid droplets came into contact with the zein solution containing the calcium chloride, the SA was crosslinked by the calcium ions in the solution to form SA/BG microspheres. Upon the evaporation of ethanol, the solubility of the zein protein decreased, leading to protein precipitation onto the surface of the SA/BG microspheres to form Zein/SA/BG microspheres. Then, the Zein/SA/BG microspheres were collected, washed, and stored at 4 °C until further use. For the control group, SA/BG microspheres without zein coating were prepared by the same method by adding SA solution containing BG into the ethanol solution containing calcium ions.

### Nile Blue Staining of the Zein/SA/BG and SA/BG Hydrogel Microspheres

To visualize the zein coating of the Zein/SA/BG microspheres, Nile blue staining was performed. The Zein/SA/BG microspheres and the SA/BG microspheres were placed into dd H_2_O and incubated with 1 mg mL^‐1^ Nile blue solution for 10 min. The solution was then removed, and the microspheres were washed with dd H_2_O for three times before they were imaged at 555 nm wavelength by confocal microscopy.

### Scanning Electron Microscopy Imaging of the Microspheres

The Zein/SA/BG and SA/BG microspheres were frozen at ‐80 °C for 2 h before they were freeze‐dried. Then, the microspheres were placed onto the aluminum stubs and sputter‐coated with an ultrathin gold layer on the surface. Afterward, the samples were visualized by using a scanning electron microscope (SEM, Hitachi S‐4800, Japan) with accelerating voltages of 10 kV. The size, shape, and surface morphology were recorded.

### Fourier‐Transform Infrared Spectroscopy (FTIR) Analysis of Microspheres

FTIR was used to identify and characterize the Zein protein in the Zein/SA/BG microspheres. The Zein/SA/BG and the SA/BG microspheres were freeze‐dried and grounded into powders (<2 µm in size) before the samples were extruded to form a thin sheet. The gas chamber was evacuated before the samples were tested. The spectrum of the Zein/SA/BG and SA/BG powders were obtained and the absorption bands were matched to the corresponding vibrational modes of various chemical groups.

### Thermogravimetric Analysis (TGA) of Microspheres

To evaluate whether Zein/SA/BG microspheres can protect BG from the acidic environment, Zein/SA/BG and SA/BG microspheres were incubated with HCl and dd H_2_O solution (control). The concentration of the HCl was adjusted to be pH = 2, which was close to the pH value of the gastric acid. After incubating Zein/SA/BG and SA/BG microspheres for 2 h with the HCl solution or dd H_2_O solution, the residual microspheres were collected and water on the surface of the microspheres was removed. The gross weight of the microspheres was then measured and recorded. Moreover, to further assess the protective effects of microspheres on the BG in the acidic environment, the weight change of BG before and after the incubation was measured with TGA. Briefly, the microspheres were gradually heated up to 900 °C to remove the zein protein and SA, leaving behind residues consisting mainly of BG. The weight of the residues before and after TGA was recorded, and the weight change was calculated.

### In Vitro Simulation of the Digestion Process

To simulate the digestion process, simulated gastric fluids (SGF) and simulated intestinal fluids (SIF) were first prepared. The simulated gastric fluid was prepared by adding 1 g of the pepsin, 1.5 g gastric mucin and 8.775 g sodium chloride into 1 L sterilized dd H_2_O and adjusting the pH of the solution to 1.3 by adding HCl solution according to a published protocol.^[^ ^56^ ^]^ The simulated intestinal fluid was prepared by adding 1 g of the pancreatin, 3 g of the bile salt and 5 g of the sodium chloride into 1 L sterilized dd H_2_O and adjusting the pH of the solution to 8.0 by adding NaOH solution according to a published protocol.^[^ ^57^ ^]^ Then, 1 g of the Zein/SA/BG and SA/BG microspheres were incubated with 10 mL of the SGF for 2 h at 25 °C. Afterward, the microspheres were collected before their images were taken, and weight was measured and recorded. The microspheres were further incubated with the SIF at 25 °C for 2 and 12 h, respectively. Similarly, images and weight of the microspheres were taken and recorded. The relative weight of the microspheres to their original weight was calculated to determine the extent of degradation of the microspheres in SGF and SIF, respectively.

### Cytotoxicity Testing of Zein/SA/BG Hydrogel Microspheres In Vitro

To evaluate the biocompatibility of Zein/SA/BG microspheres, the microspheres were directly cultured with the RAW cells. Briefly, RAW cells (1.5 × 10^6^ cells mL^‐1^) were seeded into 12 well plates before the Zein/SA/BG microspheres were added (25 mg mL^‐1^). After culture for 12, 24, 36, 48 and 60 h, the viability of RAW was tested by Cell Counting Kit‐8 assay (CCK‐8) (TargetMol, C0005). The relative cell viability was calculated.

### Evaluation of Therapeutic Potential of Zein/SA/BG Hydrogel Microspheres in acute DSS‐Induced Mice

The animal experiments were performed with the approval of the Animal Care and Experimental Committee of the Sixth People's Hospital affiliated to School of Medicine, Shanghai Jiao Tong University (Approval number: DWLL2022‐0437). Dextran sulfate sodium (DSS) (MW: 36 000–50 000) was applied to induce acute inflammation in the intestine of mice according to previous studies.^[^ ^58^, ^59^ ^]^ Twenty C57 mice were randomly divided into five groups. Mice in group A were fed with normal water and food (Control group). Mice in other four groups (B–E) were fed with 3% (W/V) DSS solution in drinking water for 4 d to induce acute colitis. Following the appearance of the symptoms of the IBD including production of stool with bloods and weight loss, the mice with colitis were randomly divided into four groups. These mice were fed with the 3% (W/V) DSS solution in drinking water to maintain the inflammation in the intestine for 7 d. During these 7 d, mice in these four groups were further treated with different microspheres through oral gavage. Mice in group B were fed with saline daily for 7 d. Mice in group C were fed with SA/BG microspheres in saline daily for 7 d. Mice in group D were fed with Zein/SA/BG microspheres in saline daily for 7 d. Mice in group E were fed with Zein‐SA‐BG mixture (by mixing the three components without forming microspheres) in saline daily for 7 d (BG dosage: 0.002 g per day for all groups). The weight of the mice in all groups was recorded daily during the whole experiment. On day 8, all mice were sacrificed, and the intestinal tissues were collected. The gross image of the large intestine was taken, and the length of the large intestine was measured. The large intestines of the mice from the five groups were fixed with formalin solution for 24 h, washed by PBS, and embedded into paraffin. The embedded tissues were sectioned into 10 µm slides with a microtome. The slides were subjected to H&E staining and imaged by an upright microscope connected to a CCD camera. To compare the therapeutic efficacy of Zein/SA/BG microspheres with that of 5‐aminosalicylic acid (5‐ASA), 12 C57 mice were randomly divided into four groups and fed with normal water and food (Control group), or given saline, BG (0.002 g per day), or 5‐ASA (200 mg per kg per day) after acute colitis is induced. The dosage of 5‐ASA was selected based on previous reports.^[^ ^60^, ^61^, ^62^ ^]^ After treatment for 7 d, all mice were sacrificed and similar analyses were performed as described above.

### Immunostaining of the Intestinal Tissues

The sectioned slides of the scaffold were dewaxed, rehydrated, and incubated with the H_2_O_2_/methanol for inactivating the endogenous peroxidase. Then, the slides were incubated with the BSA solution before incubated with the primary antibodies including anti‐ZO‐1 antibody (Servicebio, GB111402), anti‐iNOS antibody (Servicebio, GB11119), anti‐ARG antibody (Servicebio, GB11285), anti‐CD80 antibody (Bioss, BS‐2211R), anti‐CD86 antibody (Servicebio, GB13585), ani‐F4/80 antibody (Servicebio, GB11027), and anti‐*α*‐smooth muscle actin (SMA) antibody (Servicebio, GB13044). Subsequently, the slides were washed by PBS solution for three times and incubated with the secondary antibodies as well as the DAPI solution. Finally, the slides were dehydrated and mounted for imaging.

### 16S rRNA Sequencing Analysis of Intestinal Microbiota

To analyze the compositional difference of intestinal microbiota in DSS‐mice following different treatments, 16S rRNA sequencing was performed by PANOMIX. The processing steps are referenced from previous literature.^[^ ^63^ ^]^ Briefly, the feces in the intestine from different mice were collected after euthanasia. The feces were then frozen in the liquid nitrogen. The DNA of microbiota was extracted from the feces and the 16S rRNA libraries were constructed. The 16S rRNA gene sequences were analyzed by the QIIME 2 data analysis package.

### Evaluation of Therapeutic Potential of Zein/SA/BG Hydrogel Microspheres in Chronic DSS‐Induced Mice

Fifteen C57 mice were randomly divided into three groups. Chronic colitis was induced by administering three cycles of relative low concentration of DSS at 2% according to previous studies.^[^ ^64^, ^65^ ^]^ In each cycle, the mice were given 2% DSS in drinking water for 7 d and normal drinking water for 14 d. The mice in control group were given normal drinking water during the whole experiment. After three cycles of DSS feeding, the mice were given Zein/SA/BG microspheres (BG dosage: 0.002 g per day) or saline for 21 d. At the same time, the fourth cycle of DSS treatment (by 2% DSS in drinking water in the first 7 d and normal drinking water in the following 14 d) will be administered to maintain the inflammatory condition. The weight of the mice after different treatments during the fourth cycle was recorded. At the end of the experiment, the mice were sacrificed, and the large intestines were collected and imaged. They were then fixed, and sectioned. H&E staining, Masson staining, and immunostaining of *α*‐SMA and iNOS were performed. Besides, the spleen, lung, and kidney of the mice after different treatments were also collected, fixed, sectioned, and stained by H&E.

### Statistical Analysis

Statistical analysis was conducted using GraphPad Prism 9 software. The values are expressed as means ± SD. Significant differences among groups were analyzed using one‐way analysis of variance (ANOVA) followed by Tukey's test, and p<0.05 was considered as statistically significant. At least three independent experiments were performed for the in vitro cell culture experiments.

## Conflict of Interest

Y.Z. and H.F.C. are inventors of a patent being filed.

## Supporting information

Supporting Information

## Data Availability

The data that support the findings of this study are available from the corresponding author upon reasonable request.
