# Supplementary material for: Oral Delivery of Bioactive Glass‐Loaded Core–Shell Hydrogel Microspheres for Effective Treatment of Inflammatory Bowel Disease
Source: Adv Sci (Weinh). 2023 Apr 24;10(18):2207418. doi: 10.1002/advs.202207418 (PMC10288274; doi:10.1002/advs.202207418)
Supplement: Supplementary file 1 — Supporting Information [file ADVS-10-2207418-s001.pdf]

ADVANCED SCIENCE
Open Access

Supporting Information

for *Adv. Sci.*, DOI 10.1002/advs.202207418

Oral Delivery of Bioactive Glass-Loaded Core–Shell Hydrogel Microspheres for Effective Treatment of Inflammatory Bowel Disease

Yanlun Zhu, Yiwei Wang, Guanggai Xia, Xuerao Zhang, Shuai Deng, Xiaoyu Zhao, Yanteng Xu, Guozhu Chang, Yu Tao, Mingqiang Li, Haiyan Li, Xinyu Huang* and Hon Fai Chan**

Supporting Information

Title Oral delivery of bioactive glass-loaded core-shell hydrogel microspheres for effective treatment of inflammatory bowel disease

Yanlun Zhu, *Yiwei Wang*, *Guanggai Xia*, *Xuerao Zhang*, *Shuai Deng*, Xiaoyu Zhao, *Yanteng Xu*, *Guozhu Chang*, *Yu Tao*, *Mingqiang Li*, *Haiyan Li*[*], *Xinyu Huang*[*], *Hon Fai Chan*[*]

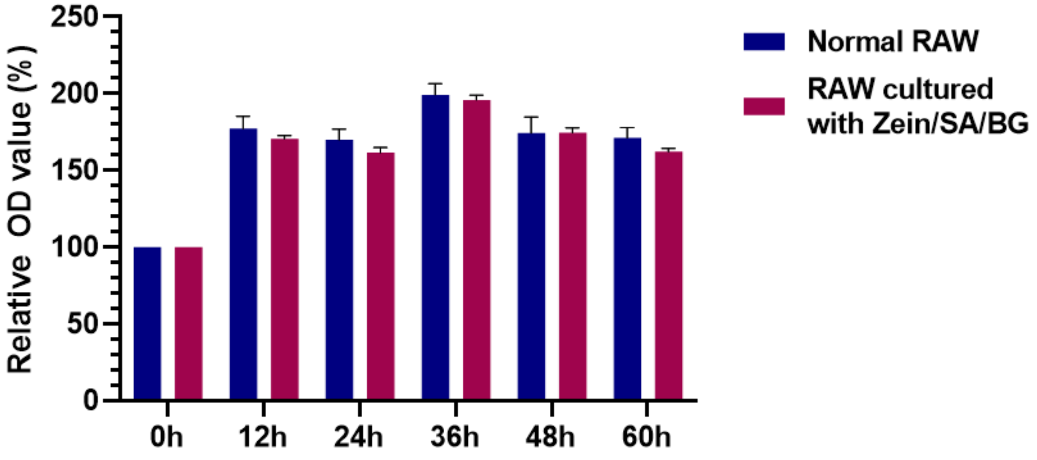

Figure S1. CCK-8 test of RAW cells cultured in the presence (RAW cultured with Zein/SA/BG) or absence (Normal RAW) of Zein/SA/BG microspheres. No significant statistical difference was detected between the two groups. (n=3)

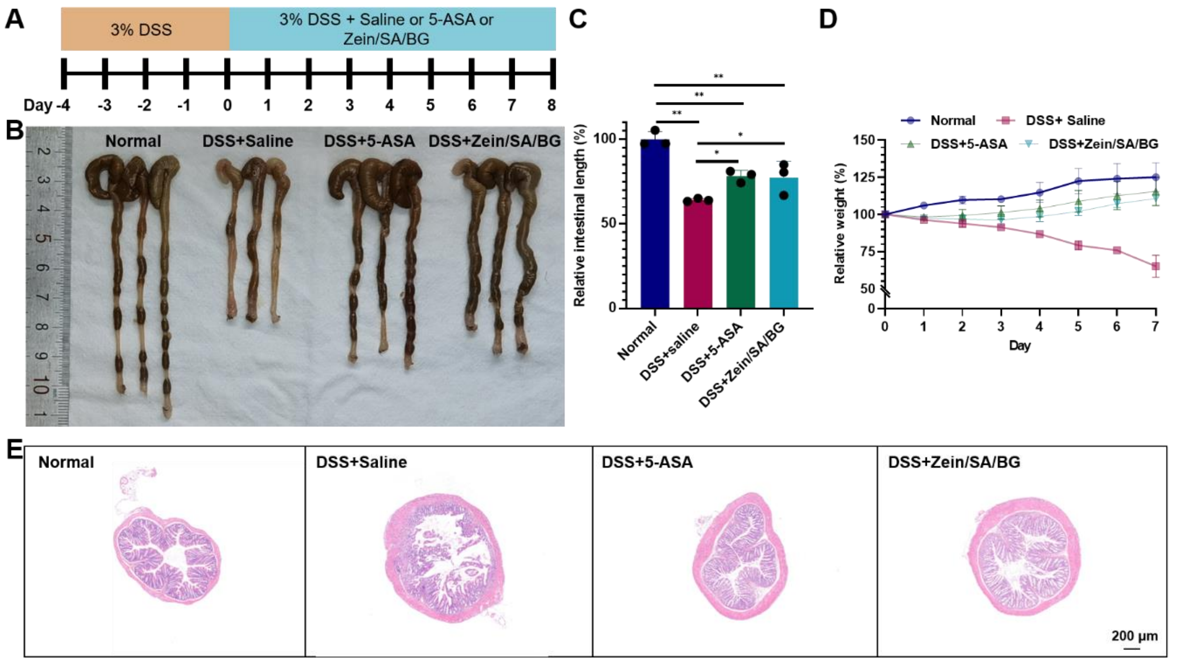

Figure S2. Therapeutic effect of 5-ASA and Zein/SA/BG microspheres in DSS-induced acute colitis mice. A) Timeline of the *in vivo* experiment. B) Gross image of the large intestine from normal mice and DSS-induced mice after different treatments. (“Normal” stands for the large intestine from normal mice; “DSS+Saline” stands for the large intestine from DSS-induced mice treated with saline; “DSS+5-ASA” stands for the large intestine from DSS-induced mice treated with 5-ASA; “DSS+Zein/SA/BG” stands for the large intestine from DSS-induced mice treated with the Zein/SA/BG microspheres) C) Relative length of large intestine of normal mice and DSS-induced mice after different treatments. D) Relative weight changes of normal mice and DSS-induced mice after different treatments. E) H&E staining of the large intestine from normal mice and DSS-induced mice after different treatments. (n=3)

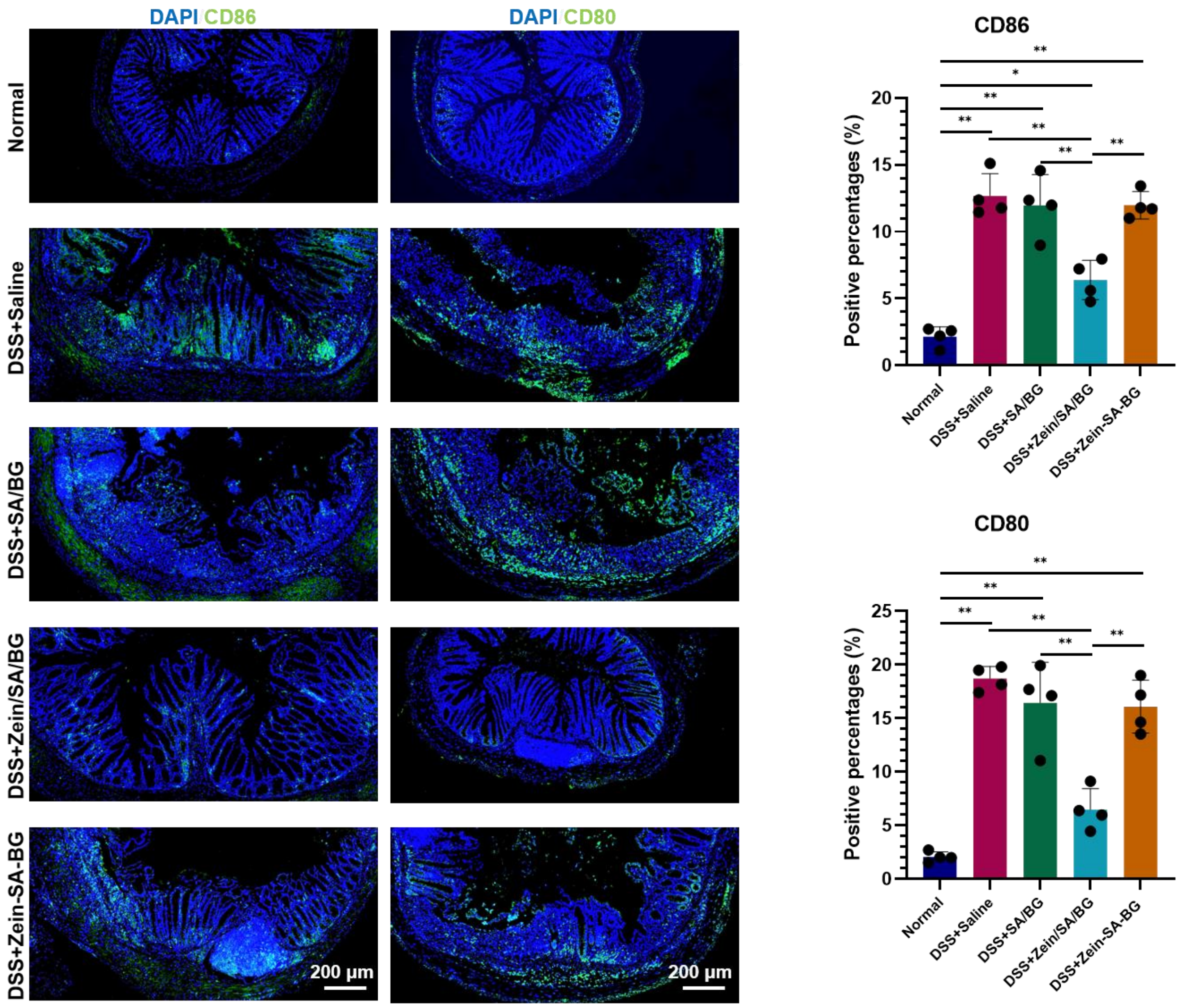

Figure S3. Immunostaining of CD86 and CD80 in the large intestine of normal mice and DSS-induced acute colitis mice after different treatments. (n=4, ** indicates p<0.01)

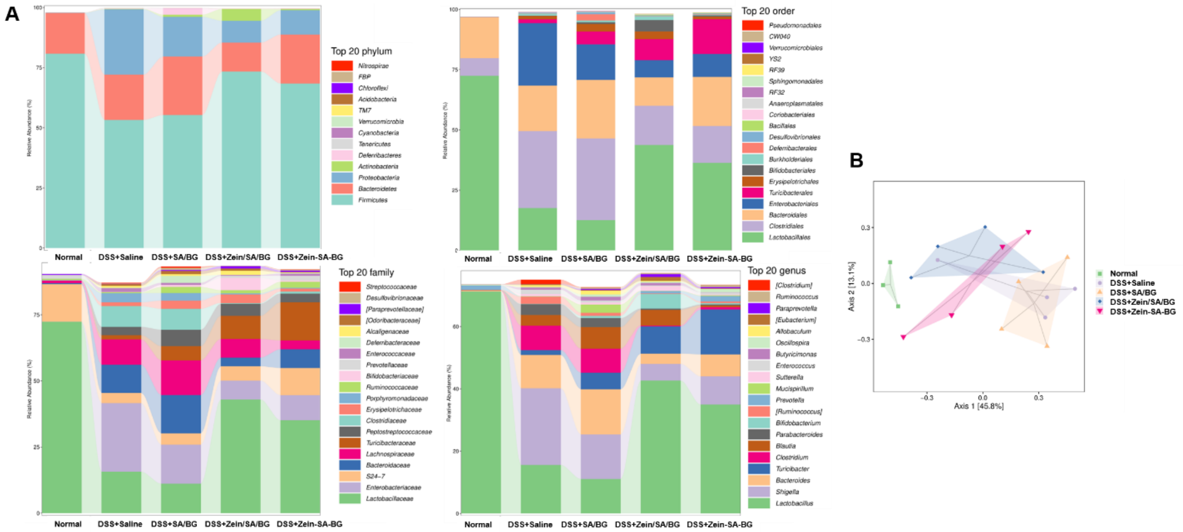

Figure S4. Effect of BG-based treatments on intestinal microbiota in normal and DSS-induced acute colitis mice. A) Distribution of intestinal microbiota species of normal mice and DSS-induced acute colitis mice after different treatments B) Principal coordinate analysis (PCoA) of intestinal microbiota from normal mice and DSS-induced acute colitis mice after different treatments.

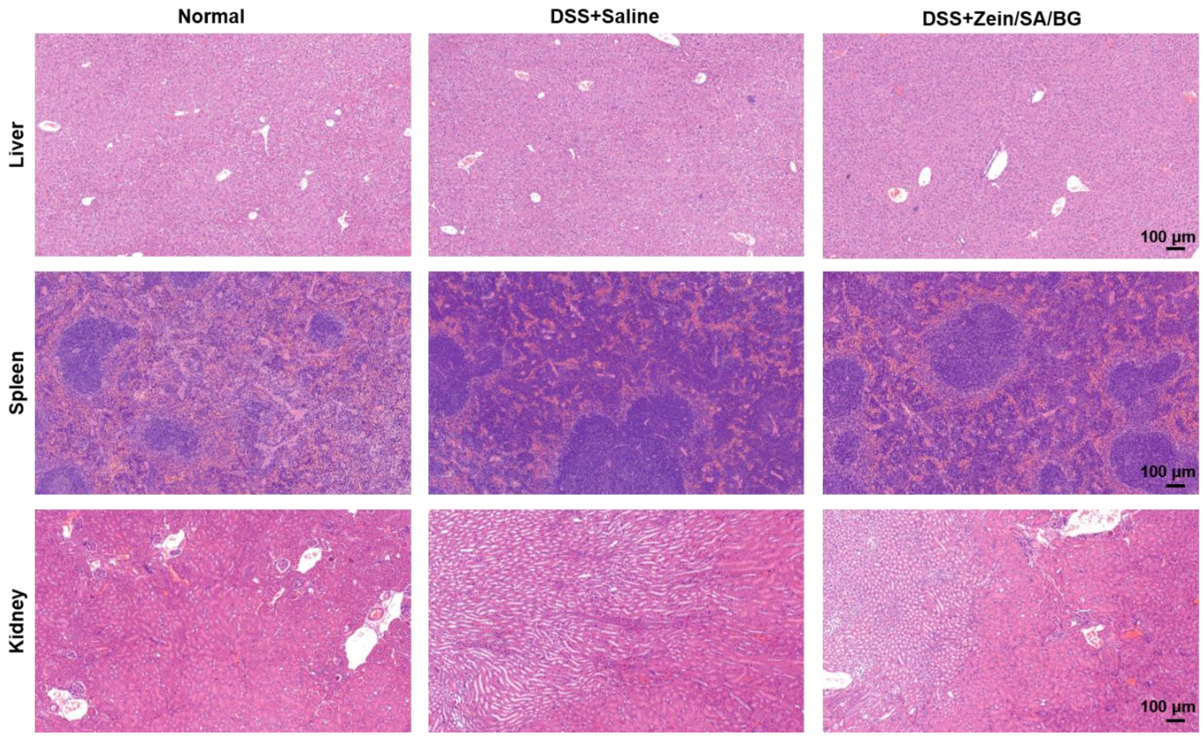

Figure S5. H&E staining of the liver, spleen, and kidney from normal mice and DSS-induced acute colitis mice after different treatments.

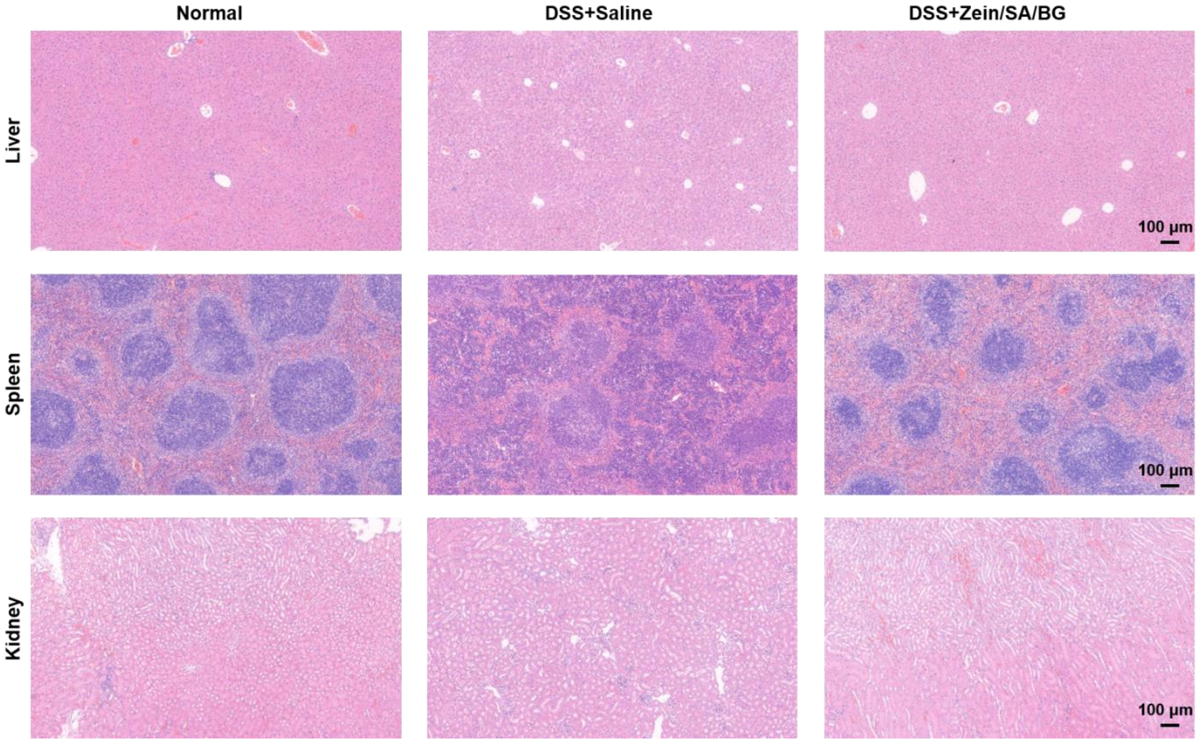

Figure S6. H&E staining of the liver, spleen and kidney from normal mice and DSS-induced chronic colitis mice after different treatments.
